# Concrete Multi-Type Defect Classification Algorithm Based on MSSMA-SVM

**DOI:** 10.3390/s22239145

**Published:** 2022-11-25

**Authors:** Xu Tian, Jun Ao, Zizhu Ma, Bijian Jian, Chunbo Ma

**Affiliations:** 1 Research Institute of Optical Communication, School of Information and Communication, Guilin University of Electronic Technology, Guilin 541004, China; 2 Pengcheng Laboratory, Shenzhen 322099, China

**Keywords:** concrete defect classification, wavelet packet transform, laser vibration measurement technique, support vector machine, slime mold algorithm

## Abstract

In order to realize the automatic classification of internal defects for non-contact nondestructive testing of concrete, a concrete multi-type defect classification algorithm based on the mixed strategy slime mold algorithm support vector machine (MSSMA-SVM) was proposed. The concrete surface’s vibration signal was obtained using a laser Doppler vibrometer (LDV) for four classification targets for no defect, segregation, cavity, and foreign matter concrete classification targets. The wavelet packet transform (WPT) decomposes the detected signals to get information on different frequency bands. The energy ratio change rate, energy ratio, and wavelet packet singular entropy of each node after the WPT were used as the feature input of MSSMA-SVM. The experimental results show that the designed MSSMA-SVM classifier can accurately detect the type, which provides a practical algorithm for classifying concrete defects by laser vibration measurement.

## 1. Introduction

Detecting internal defects in concrete has been a hot issue in construction engineering disaster research. During the production and service of concrete, the structure may deteriorate due to human or external environmental influences, which leads to the deterioration of concrete’s load-bearing capacity, sealing performance, and water resistance. Currently, standard concrete non-destructive testing (NDT) inspection methods include the ground penetrating radar method [1,2,3], infrared thermography [4,5,6], ultrasonic detection [7,8,9], and so on. However, the above NDT methods have certain limitations in practical applications. For example, a ground-penetrating radar needs to be close to the measured target in the test, and when the test site is complicated, it will affect the normal use of a ground-penetrating radar. In addition, the electromagnetic wave signal of a ground-penetrating radar is easily shielded by the steel mesh, which is less effective in detecting the concrete structure containing the steel mesh. Infrared thermography detection results are susceptible to external temperature or heating time, and it is not easy to locate the depth of the defects. Ultrasonic detection requires contact with the object. Its detection effect is susceptible to the interference of the transducer and object surface coupling state, which is challenging to apply to detect irregularly shaped objects. Laser vibrometry is a non-contact NDT technique with high detection bandwidth and spatial resolution advantages. Compared with conventional contact detection, laser vibration measurement avoids the influence of additional mass and stiffness of the sensor on the structure, skips the anchoring and wiring process of the sensor, shortens the test time, and reduces measuring errors. It has been widely used in many primary scientific fields [10,11,12]. Laser vibration measurement technology has recently been applied to concrete defect detection. For example, Yasuda et al. induced vibrations on the surface of concrete tunnels by laser ablation and used laser vibrometry to measure vibration signals to detect the health of tunnel linings [13,14]. Using remote directional acoustic radar as the sound generation source, Sugimoto et al. investigated a method to detect internal defects in underground tunnels and viaducts by laser vibrometry [15,16]. However, although the methods mentioned above can effectively distinguish between abnormal and normal areas inside the concrete, it is difficult to effectively classify the types of typical defects in concrete, such as segregation, cavities, foreign matter, and so on. Segregation refers to separating concrete raw materials from each other, generally caused by over-drying or over-diluting raw materials during mixing. The segregation defect causes poor homogeneity of the concrete, affecting the load-bearing capacity of the concrete structure and the durability of the concrete, due to seepage and frost resistance, for example. A cavity refers to a hole within the concrete structure that exceeds the thickness of the concrete structure’s protective layer in depth and length. The cavity defect is caused by concrete blockage or poor pounding when filling some parts, which can easily cause structure breakage or destroy the structure’s integrity. Foreign matter defect refers to the mix of debris inside the concrete structure, which can lead to structure strength loss. Therefore, the classification of defect signals is significant because it is difficult to assess the health of buildings and hazard warnings without knowing the types of defects, not to mention the repair and maintenance of buildings. In addition, although a large number of studies have been conducted by a majority of scholars on the problem of concrete defect classification and many good results have been obtained [17,18,19,20,21,22], most use contact non-destructive detection methods. Considering that the laser vibrometer signals may differ from those collected by contact sensors, it is necessary to study an automated identification algorithm for internal concrete defects based on laser inspection.

Unlike traditional ultrasonic inspection methods, this paper uses a contact ultrasonic transducer for acoustic vibration excitation on the concrete surface and an LDV to measure the vibration velocity signal of the concrete surface to achieve non-contact measurement. For the characteristics of vibration signals of different types of defects, the nodal energy ratio variation rate, nodal energy ratio, and wavelet packet singular entropy constituting feature parameters were extracted by WPT and input to the SVM classifier for training. In addition, to improve the accuracy of the SVM model recognition, the MSSMA is proposed to optimize the SVM learning parameters to improve the accuracy of the SVM for defect classification.

## 2. Theoretical Description and Algorithmic Steps

### 2.1. LDV Measurement Principle

The Doppler effect is the basis for an LDV to measure the vibration velocity of the surface of the measured object. During the measurement, the laser from the laser generator inside the LDV scanning head is divided into two identical laser beams after passing through the beam-splitting prism, assuming that their frequencies are both *f*_0_. One of the lasers directly hits the photodetector as the reference beam. The other laser is emitted from the scanning head’s laser beam aperture as a detection beam, focused on the measured object’s surface and received by the photodetector after reflection. Due to the Doppler effect, the slight vibration of the object’s surface causes the frequency of the reflected detection beam to change. Assuming the reflected detection beam frequency is *f*_1_, the vibration speed of the measured object surface is obtained according to the following formula for the Doppler shift.

(1) $$\Delta f=f_{1}-f_{0}=\frac{2v\cos\theta }{\lambda }$$

where Δf denotes the Doppler shift of the detection beam. v denotes the vibration velocity of the measured object surface. θ denotes the angle between the direction of incidence of the detection beam and the normal direction of the measurement point, and vcosθ denotes the vibration velocity component of the object surface perpendicular to the direction of detection beam incidence. λ represents the wavelength of the laser.

### 2.2. Feature Extraction

To make full use of the time-frequency information of ultrasonic waves, we extracted three kinds of features based on wavelet packet theory [23] to characterize the measurement signals of different defect types:

- Nodal energy ratio variation rate;
- Nodal energy ratio;
- Wavelet packet singular entropy.

#### 2.2.1. Nodal Energy Ratio Variation Rate

The nodal energy ratio variation is a measure of the difference in energy between the wavelet packet nodes of two signals and is defined as follows.

(2) $$\mathrm{WPER}=\sqrt{\sum_{i=0}^{2^{j}-1}{\left({\mathrm{WPE}}_{i}-\bar{\mathrm{WPE}}\right)}^{2}}$$

where ${\mathrm{WPE}}_{i}$ denotes the rate of change of the wavelet package energy of the *i*-th node signal in layer *j*. $\bar{\mathrm{WPE}}$ denotes the average of the wavelet package energy change rates of all node signals in layer *j*.

${\mathrm{WPE}}_{i}$ is defined as follows.

(3) $${\mathrm{WPE}}_{i}=\left|\frac{{\mathrm{WP}}_{i}^{\mathrm{RS}}}{\left(\sum_{i=0}^{2^{j}-1}{\mathrm{WP}}_{i}^{\mathrm{RS}}/2^{j}-1\right)}-\frac{{\mathrm{WP}}_{i}^{\mathrm{MS}}}{\left(\sum_{i=0}^{2^{j}-1}{\mathrm{WP}}_{i}^{\mathrm{MS}}/2^{j}-1\right)}\right|$$

where ${\mathrm{WP}}_{i}^{\mathrm{RS}}$ and ${\mathrm{WP}}_{i}^{\mathrm{MS}}$ denote the wavelet packet energy of the reference signal and the comparison signal at the *i*-th node under layer *j*, respectively.

#### 2.2.2. Nodal Energy Ratio

The node energy ratio represents the proportion of the wavelet packet signal in each frequency domain segment.

(4) $$R_{j}^{}=\frac{E_{j}^{}}{E}=\frac{\sum_{m=1}^{N_{L}}{\left|z_{m}^{}\right|}^{2}}{\sum_{j=0}^{2^{L}-1}\sum_{m=1}^{N_{L}}{\left|z_{m}^{}\right|}^{2}}$$

where *L* stands for the number of wavelet packet decomposition layers. *z_m_* and *N*_L_ denotes the amplitude of the reconstructed signal and the number of sample points, respectively.

#### 2.2.3. Wavelet Packet Singular Entropy and Pearson Correlation Coefficients

Wavelet packet singular entropy is a signal analysis method based on singular value decomposition theory. The approach decomposes the node signal after wavelet packet decomposition reconstruction into singular values that can reflect the characteristics of the original signal and then characterizes the original signals using the statistical properties of information entropy. Since the acquired signals may contain noisy information, we eliminate some low-correlation node signals by Pearson correlation coefficients before calculating the wavelet packet singular entropy to reduce noise interference. The Pearson correlation coefficient is calculated as follows.

(5) $$\rho_{Q,O}=\frac{Cov(Q,O)}{\delta_{Q}\delta_{O}}=\frac{\sum_{i=1}^{n}\left(q_{i}-\bar{Q}\right)\sum_{i=1}^{n}\left(o_{i}-\bar{O}\right)}{\sqrt{{\sum_{i=1}^{n}\left(q_{i}-\bar{Q}\right)}^{2}{\sum_{i=1}^{n}\left(o_{i}-\bar{O}\right)}^{2}}}$$

where *Q* and *O* represent the node signal and the original signal, respectively. *q_i_* and *o_i_* denote the sampling point amplitude of the node signal and the original signal, respectively. If $\rho_{Q,O}\geq 0.3$, the singular entropy of the node signal will be calculated according to the singular entropy formula in the literature [24]. Otherwise, the singular entropy of the node signal will be set to 0.

### 2.3. SVM Defect Classification Combined with Intelligent Optimization Algorithm

There are many types of concrete. For example, concrete can be classified as cement concrete, bituminous concrete, and polymer concrete, among others, by their binding materials; according to the strength grade, the concrete is classified into c20 concrete, c30 concrete, c40 concrete, and so on. Therefore, it is challenging to establish a complete sample library of concrete defects. In particular, c30 cement concrete was used for the study in this paper. In addition, we found that some collected sample signals were too similar among the same type of test blocks, so we finally selected some representative signals as training samples (280 signal samples in total) to ensure training effects and save computational costs. Given the excellent generalization ability of an SVM for pattern recognition, classification, and regression prediction problems with limited samples, we use an SVM to build a defect recognition model.

An SVM’s commonly used kernel functions are linear function, polynomial function, radial basis function (RBF), and Sigmoid function. Since the radial basis kernel function can effectively reduce the computational complexity and requires fewer hyperparameters to be adjusted [25], the RBF is chosen as the classifier kernel function in this paper.

During the training process, the penalty factor *C*_p_ and hyperparameter γ of the SVM determines the tolerance of sample classification errors and the distribution state of the samples after mapping to the feature space, respectively. So, their values can impact the learning and generalization ability of the SVM model. However, searching for the optimal parameters is inefficient in the original SVM algorithm. To improve the accuracy of the support vector machine in identifying defects, we propose the MSSMA algorithm to optimize the parameter settings of the support vector machine and the model’s classification performance.

### 2.4. Mixed Strategy Slime Mold Algorithm (MSSMA)

The SMA [26] is a new metaheuristic optimization algorithm proposed in recent years with a robust global exploration capability. However, the algorithm has a weak oscillatory effect in the late iteration and a weak contraction mechanism, so it has the problems of easily falling into local optimum and slow convergence. To address the above issues, we make the following improvements to the SMA.

#### 2.4.1. Improved Population Initialization Strategy

The location initialization affects the diversity of the population and the quality of the algorithm’s search performance. In the population initialization phase, the search range of the SMA depends entirely on randomness, which makes it difficult to guarantee the dispersion of population locations during initialization. To improve the diversity of the initial population, we replace the initialization method of the SMA with Sobol sequence initialization [27], which makes the initial population distribution more dispersed and uniform in location. The sequences are mapped to the search interval of the optimization problem by the following equation.

(6) $$X_{J,D}(1)=SQ_{J,D}(1)\cdot (UB-LB)+LB$$

where $X_{J,D}(1)$ denotes the initialized position of the population, $SQ_{J,D}(1)$ denotes the Sobol sequence. *UB* and *LB* denote the upper and lower bounds of the problem search region, respectively. *J* and *D* denote the individual’s position and current dimension, respectively.

#### 2.4.2. Improved Population Individual Selection Strategy

To increase the diversity of the population, the MSSMA adds variation, crossover, and selection operations after population initialization and individual location updates.

(7) $$M_{J}(t+1)=X_{r1}(t)+F_{r}\cdot (X_{r2}(t)-X_{r3}(t))$$

(8) $$C_{J,D}(t)=\left\{ \begin{array}{l} M_{J,D}(t)\;,\;rand<C_{r}\;\mathrm{or}\;D=D_{rand}\\ X_{J,D}(t)\;,\;\mathrm{otherwise} \end{array}\right.$$

where *X*, *M*, and *C* denote the parent population, intermediate population, and offspring population, respectively. *F*_r_ is the scaling factor taken in the interval [0.2, 0.8] in the experiment; *r*_1_, *r*_2_, and *r*_3_ are random integers in the interval [1, *N*]; *N* is the population size; *C*_r_ denotes the crossover probability set to 0.5 in the experiment; and *rand* denotes the random number in the interval [0, 1]. In the selection operation, all offspring individuals are ranked in terms of fitness together with all parent individuals. The top *N* individuals are selected to be retained in the next generation according to their fitness.

#### 2.4.3. Improved Location Updates Strategy

The equation for updating the individuals’ position in the original SMA is as follows.

(9) $$X_{J}(t+1)=\left\{ \begin{array}{l} rand\cdot (UB-LB)+LB,rand_{1}<Z_{\mathrm{SMA}}\\ X_{\mathrm{best}}(t)+v_{b}\cdot (W\cdot X_{R1}(t)-X_{R2}(t)),rand_{2}<p\\ v_{c}\cdot X(t),rand_{2}\geq p \end{array}\right.$$

where $X_{\mathrm{best}}$ denotes the position of the individual with the best adaptation in the current iteration; *X*_*R*1_ and *X*_*R*2_ denote the positions of two different random individuals; $v_{b}$ denote a random number within a specific interval controlled by a decay factor; $v_{c}$ is a linearly decreasing parameter from 1 to 0, and *W* represents the weight coefficient; and *Z*_SMA_ is a custom parameter (value of 0.03). From the Equation (9), it is clear that at the beginning of the SMA iteration, the synergistic effect of $v_{b}$ and $v_{c}$ makes individuals move closer to the optimal position while separating some individuals to explore other domains. When the random number is less than *Z*_SMA_, the slime position will also be initialized randomly. Thus, the multiple exploration mechanism gives the SMA a powerful global optimization-seeking capability. However, the oscillatory effect of $v_{b}$ is substantially weakened in the late iteration, making it difficult for individuals to jump out of the local optimum in the late iteration. In addition, the contraction mechanism of $v_{c}$ control is weak, which tends to make the algorithm fall into premature convergence.

To address the shortcomings of the original SMA, we replace the fixed parameter *Z*_SMA_ with the linear decay factor *Z*_MSSMA_, which enhances the population diversity at the beginning of the iteration and prevents the algorithm from converging too fast to falling into premature convergence in the early iteration. In addition, the spiral search strategy with exponentially decreasing weights is used instead of the contraction strategy in the original algorithm to balance the global search ability and local search ability. Among them, the setting of the exponentially decreasing strategy factor w refers to the literature [28]. The position update formula of the MSSMA is as follows.

(10) $$X_{J}(t+1)=\left\{ \begin{array}{l} rand\cdot (UB-LB)+LB,rand_{1}<Z_{\mathrm{MSSMA}}\\ X_{\mathrm{best}}(t)+v_{b}\cdot (W\cdot X_{R1}(t)-X_{R2}(t)),rand_{2}<p_{}\\ \left|X_{\mathrm{best}}(t)-X_{R2}(t)\right|\cdot e^{v_{\mathrm{MSSMA}}}\cdot \cos(2\pi v_{\mathrm{MSSMA}})+w\cdot X_{\mathrm{best}}(t),rand_{2}\geq p \end{array}\right.$$

(11) $$Z_{\mathrm{MSSMA}}=0.3\cdot (1-t/t_{\max})$$

(12) $$v_{\mathrm{MSSMA}}=\{rand\cdot [(-1+t\cdot (-1/t_{\max})]-1\}+1$$

(13) $$w=0.4\cdot {\left(\frac{9}{4}\right)}^{1/(1+10\cdot (t/t_{\max}))}$$

The MSSMA algorithm steps are as follows:

1. Set the number of population individuals *N* and the maximum number of iterations *t*_max_;
2. Initialize the parent population location using Sobol sequence;
3. Perform mutation and crossover operations on the parent population to generate offspring;
4. Calculate the fitness of all individuals in parent and offspring populations, sort the fitness values of all individuals in parent and offspring populations in ascending order, and select the top *N* outstanding individuals;
  Update the weights *W* and linear decay factors *Z*_MSSMA_;
5. Select the update equation to update the location of the population based on the conditional threshold;
6. Update the parameters *p*, $v_{b}$ and $v_{\mathrm{MSSMA}}$.
7. Determine whether the termination condition is satisfied, and output the optimal global solution of the search if the condition is satisfied. Otherwise, return to step 3 to continue the calculation.

### 2.5. The Flow of MSSMA-SVM Algorithm

The MSSMA-SVM algorithm is divided into three main phases:

1. Wavelet packet decomposition is performed on the training samples, and the original signal is decomposed into sub-signals of different frequency bands. The features are extracted by the feature extraction method described in Section 2.2 as training samples;
2. The training samples are input into the SVM model for training, and the learning parameters of the SVM are adjusted using the MSSMA algorithm during the training process;
3. Feature extraction is performed on the test set by the same method, and the test samples are input to the trained SVM model for classification tests to obtain classification results.

## 3. Experimental Environment and Testing

### 3.1. Fabrication of Experimental Test Blocks

The sample blocks in the experiment were made of c30-class concrete. Four types of concrete sample blocks, defect-free, segregated, cavity, and foreign matter, were made according to the typical concrete defects in real life to verify the classification performance of the proposed algorithm for multiple defects. These blocks were constructed using a customized architectural structure of mixed ordinary Portland cement type I, water, sand, and gravel at 461, 175, 512, and 1252 kg/m^3^, respectively. The finished block measured 30 cm in length, 15 cm in width, and 15 cm in height. After the test blocks are manufactured, the blocks are maintained for 28 days by natural maintenance.

**Figure 1:**
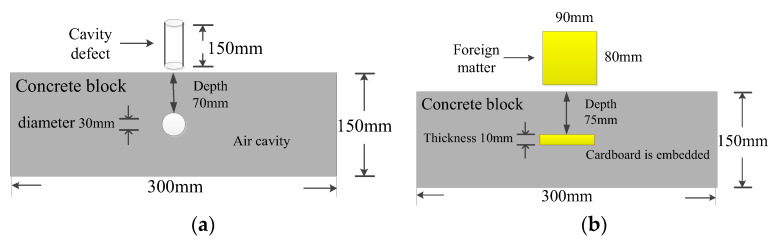
(**a**) Shape and embedded depth of cavity defect; (**b**) Shape and embedded depth of foreign matter defect.

In particular, the block that simulated a segregation defect added three times more water than the other types of test blocks. The block that simulated a cavity defect had a 3 cm diameter through-cylindrical defect in its middle position. The block that simulated foreign matter defect had a square cardboard of 8 cm in length, 1 cm in width, and 9 cm in height placed in its middle position. The specific spatial locations of the cavity and foreign matter defects are shown in Figure 1.

### 3.2. Experimental Environment and Test

The experimental system consists of an ultrasonic transducer, an LDV, and a computer. At the transmitting end, the ultrasonic transmitting controller outputs a square wave pulse signal of ±120 V to actuate a non-metal detecting ultrasonic transducer (P28F) with a center frequency of 25 kHz to generate the ultrasonic signal. The pulse emission period is 10 ms. At the receiving end, the PSV-400 LDV from Polytec is used to collect the vibration signal of the concrete surface. Since the concrete surface is poorly reflective, we paste reflection tape at the measurement point to improve the reflection strength of the signal in the experiment. All algorithms run on a computer with a 64-bit Windows operation system in which CPU specification is 2.6 GHz, Inter Core i7-6700Q with six cores, and the memory specification is 16 GB 2400 MHz DDR4, and using Matlab (R2019B).

**Figure 2:**
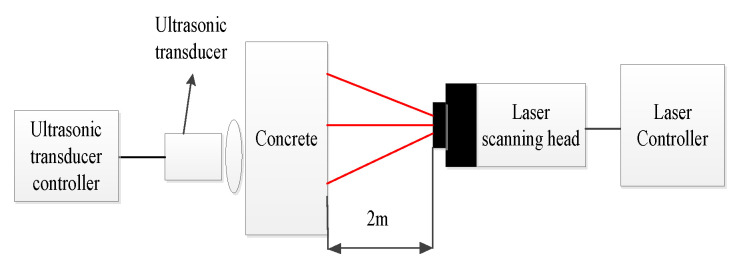
Experimental platform setup.

**Figure 3:**
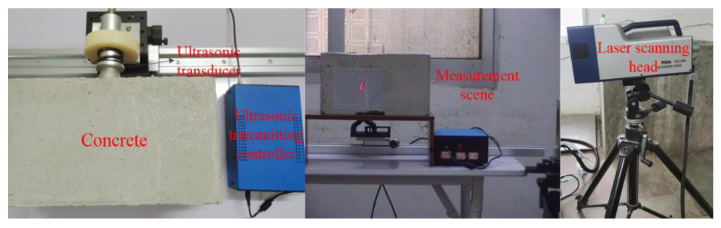
Laboratory equipment.

The experimental platform and laboratory equipment are shown in Figure 2 and Figure 3, respectively.

In the experiment, we made 16 concrete blocks for four types (4 blocks of each type). A total of 520 measurement signals were collected. In particular, 280 measurement signals were used as a training set (each type has 70 signals), and 240 were used as a test set (each type has 60 signals). The LDV sampling frequency was 125 kHz, and the sampling time was 32 ms. Each time domain signal included three complete cycles and one incomplete cycle with 4000 sampling points. In the feature extraction, only one cycle signal was extracted, with a total of 800 sampling points (the pre-triggered sampling points were ignored).

## 4. Experimental Results and Analysis

### 4.1. Measurement Signal Analysis

**Figure 4:**
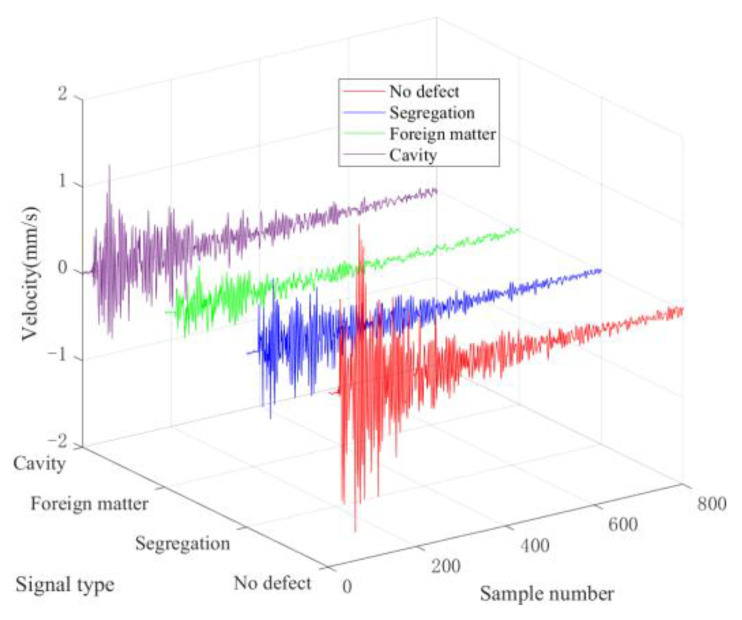
Four typical types of concrete waveforms.

Figure 4 shows the typical waveform samples randomly selected from four types of concrete detection signals collected by LDV.

As shown in Figure 4, the non-defective concrete waveform is relatively smooth, while the waveforms of the other three types of defective signals undergo different degrees of distortion. In addition, the amplitude of the defect signal is decreased compared with the no-defect signal because the influence of defects causes different degrees of attenuation, scattering, and bypassing during the propagation of ultrasonic waves. However, visually distinguishing the types of defect signals in the time domain is challenging.

**Figure 5:**
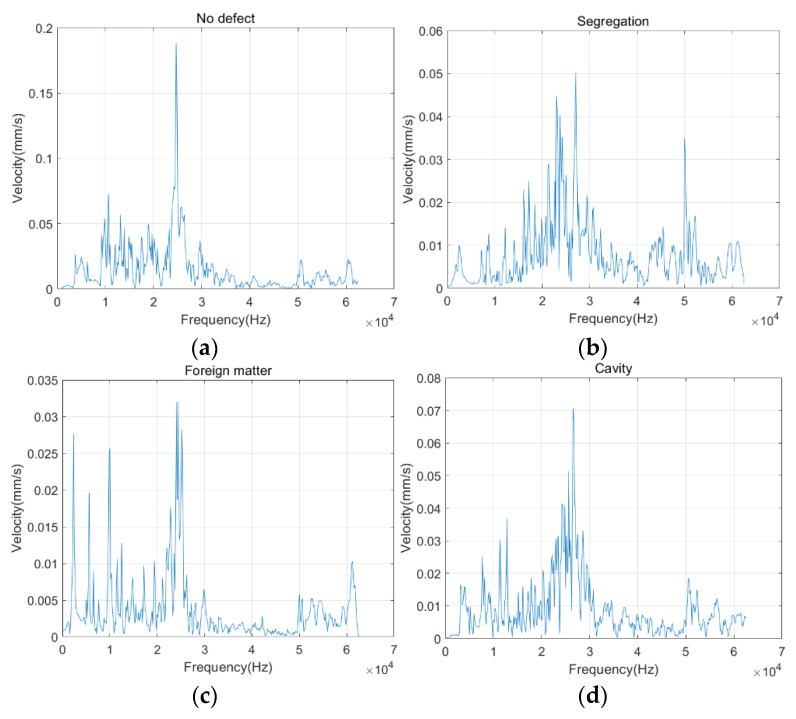
(**a**) No defect typical frequency spectrum; (**b**) Segregation typical frequency spectrum; (**c**) Foreign matter typical frequency spectrum; (**d**) Cavity typical frequency spectrum.

Figure 5 shows the spectrum of the above typical signal after the Fourier transform. It is clearly visible that the four different detection signals had similarities and differences. The spectra of all four types of signals have a distinct vibration peak around 25 kHz, caused by the ultrasound’s central frequency. In addition, the spectrum of the concrete signal without defects presents a single peak. In contrast, the spectrum of the other three defective signals contains multiple vibration peaks located at different frequencies and have different vibration amplitudes.

**Figure 6:**
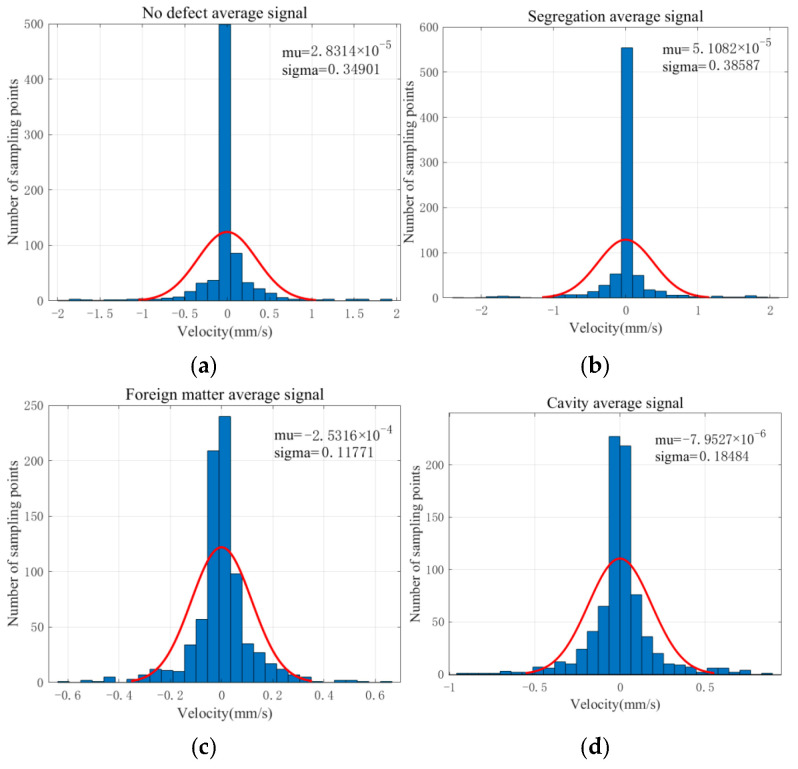
(**a**) Velocity amplitude histogram of no defect average signal; (**b**) Velocity amplitude histogram of segregation average signal; (**c**) Velocity amplitude histogram of foreign matter average signal; (**d**) Velocity amplitude histogram of cavity average signal.

To further analyze the signal characteristics of the above four types of concrete, we calculated the corresponding average signals according to the four types of defect signal training sets, then obtained a velocity amplitude statistics histogram of the average signal, as shown in Figure 6. The abscissa indicates the amplitude of the vibration velocity, the ordinate indicates the count value of the signal sampling point in the same amplitude ranges, and the red curve shows the approximate Gaussian distribution curve of the histogram.

As seen from Figure 6, the average signals of the four types all approximately obey a normal distribution. However, there are apparent differences in the standard deviation. In addition, the maximum amplitude of the signals of foreign matter and cavity is smaller than that of the non-defective and segregation.

**Figure 7:**
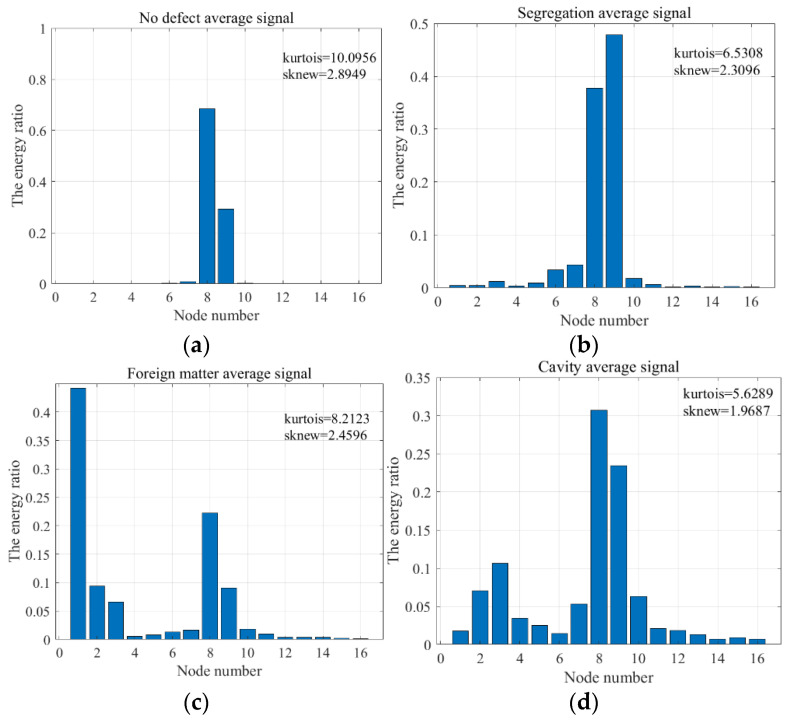
(**a**) Wavelet packet energy ratio of no defect average signal; (**b**) Wavelet packet energy ratio of segregation average signal; (**c**) Wavelet packet energy ratio of foreign matter average signal; (**d**) Wavelet packet energy ratio of cavity average signal.

On the other hand, the wavelet packet transform was used to decompose the four types of average signals into four layers. Then we obtained the energy ratios of 16 different frequency band components and calculated the kurtosis and skewness coefficients of the energy distribution, as shown in Figure 7. The kurtosis coefficient is a statistical parameter used to characterize the peak height of the data distribution curve. The skewness coefficient is a statistical parameter characterizing the degree of data asymmetry.

Figure 7 shows that the energy distribution of all four types of signals is right-skewed (skewness > 0), but the degree of skew is different. Among them, the kurtosis coefficient of the non-defective signal type is the largest, indicating that its energy is most concentrated and mainly distributed in nodes 8 and 9. In contrast, the energy distribution of the three defective signal types is more dispersed. The separation signal type and the cavity signal type’s energy distributes in different nodes. The foreign matter signal type’s energy concentrates in the low frequency, and its main frequency band locates in the first node. In summary, the four types of detection signals have significantly different time-frequency information. Therefore, we can characterize the differences between the different defect signals according to the feature extraction method in Section 2.2. 

### 4.2. Algorithm Performance Analysis

#### 4.2.1. The Effect of Decomposition Layers

This subsection tests the effect of different wavelet packet decomposition levels on the recognition performance of the algorithm. Considering that Symlet wavelets have better symmetry while retaining the tight support and smoothness of Daubechies wavelets [29]. We selected sym20 as the basis function and tested the correct recognition rate under 2~5 layers of wavelet packet decomposition. The SVM parameters *C*_p_ and γ were set to fixed values (*C*_p_ = 1, γ = 0.5). Table 1 shows the recognition correctness of the SVM model with different wavelet packet decomposition layers.

L2~L5 indicate the feature extraction using 2~5 layers of wavelet packet decomposition, respectively. Data1 to Data4 represent the test sets for no defect, segregation, foreign matter, and cavity. Comparing L2~L4, it is clear that the classification correct rate keeps increasing with the increase of wavelet packet layers. It indicates that the multi-layer wavelet packet decomposition makes the band division of each node signal more refined, and the node signal effectively expresses the characteristics of the original signal. However, when the number of wavelet packet decomposition layers is five, the correct rate of defect recognition decreases, indicating that the signal is over-decomposed. The information in each node signal after over-decomposition is too little, making it difficult for the extracted features to characterize the original signal accurately. Therefore, this paper uses four-layer wavelet packet decomposition after balancing the complexity and recognition effect.

#### 4.2.2. Impact of Features on Classification Performance

In this subsection, different learning features are tested to evaluate the proposed joint features’ impact on the model’s classification performance. Feature 1 represents the classification learning using nodal energy ratio variation rate alone. Similarly, feature-2 and feature-3 indicate that node energy ratio and wavelet packet singular entropy alone are used for classification learning. Feature-all indicates that the three combined features are used for classification learning. The model parameters settings are the same as in Section 4.2.1. Table 2 shows the recognition correctness of the SVM model with various features.

As seen from Table 2, the nodal energy ratio variation rate as a separate learning feature is difficult to distinguish multiple types of defects effectively, and the total recognition correct rate is only 34.17%. Node energy ratio and wavelet packet singular entropy achieved relatively good classification results, with overall recognition correct rates of 88.83% and 81.25%, respectively. The total recognition accuracy of the joint features was the highest, with 56.66%, 2.00%, and 9.58% improvement compared with feature-1, feature-2, and feature-3, respectively, which confirmed the effectiveness of the proposed joint features.

#### 4.2.3. Performance Comparison of Similar Algorithms

In this subsection, we compare the performance improvement of GWO [30], WOA [31], MFO [32], SMA, and MSSMA on the same dataset for SVM classification performance to verify the superiority of the proposed model among similar algorithms. The classification results of different models are shown in Table 3. The initial parameter settings of all optimization algorithms were the same, the number of populations was 20, the maximum number of iterations was 30, and *C*_p_ and γ were searched in the range of [0.0002, 5000]. The other parameter settings of the intelligent optimization algorithm were used with their respective default parameters. Each model runs 500 times independently to reduce randomness errors. The mean and standard deviation of the correct recognition rate was used to evaluate the performance of the models.

Table 3 shows that the correct rates of the SVM defect recognition models optimized by the intelligent optimization algorithm are all higher than those of the models with default parameters. It indicated that the intelligent optimization algorithm could help the SVM search for the optimal learning parameters more accurately and thus better obtain the optimal solution of the classification problem in the feature space. In addition, the AVG and STD of the total recognition rate of MSSMA-SVM are 96.29% and 0.18, respectively, which is the best performance among all models. It illustrates that the parameter search capability of the proposed MSSMA algorithm in this defect classification model is better than other intelligent optimization algorithms, which helps the SVM to identify these concrete defect signals better.

**Figure 8:**
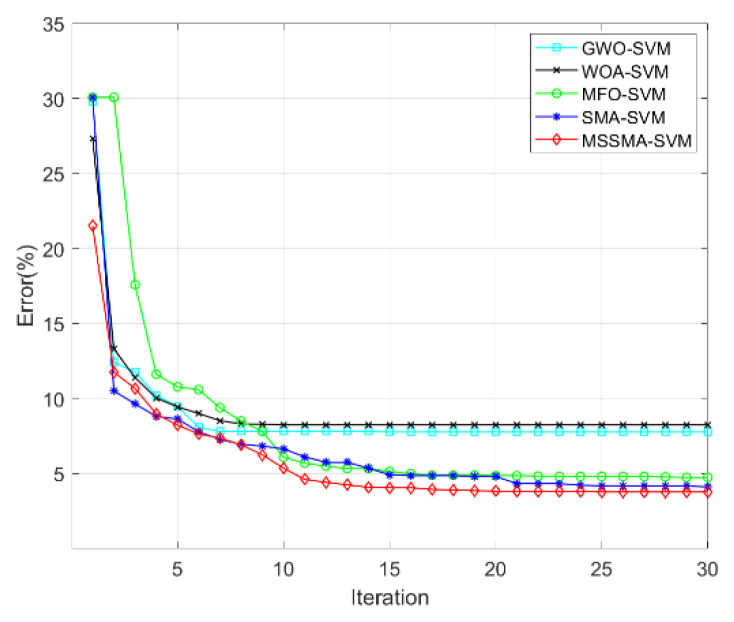
Error curves of different models.

Figure 8 shows the error convergence curves during the iterative computation of each model. After improving the population initialization strategy, the MSSMA-SVM can obtain a higher recognition correct rate than other models at the beginning of the iteration. At the same time, the MSSMA enhances the local exploration ability while retaining the global search ability due to the integration of the differential evolution strategy and the spiral search strategy, which enables it to obtain the highest recognition correct rate at the end of the iteration.

#### 4.2.4. Performance Comparison with Existing Algorithms

In order to verify the defect recognition capability of the proposed algorithm among different supervised learning algorithms, we evaluated the classification performance of an MSSMA- SVM, a BP, an RF, and an SCN on the same dataset. The algorithms that participated in the comparison were used with default parameter settings. All algorithms were run individually 500 times to reduce the effect of random factors in the algorithms on the results. The parameter settings of each algorithm are shown in Table 4. We calculated the average accuracy based on the accuracy of each test.

Table 5 shows that the recognition rates of the above models for different types of concrete defect signals are above 90%, indicating that the proposed joint features can better reflect the relevant features of concrete defect signals. In addition, the average accuracy of the MSSMA-SVM is better than other supervised learning algorithms. The reason is that in building a multi-type defect recognition model, the algorithms involved in the comparison may have problems with an unreasonable network structure and parameter settings, affecting the algorithm’s recognition accuracy during testing. At the same time, the MSSMA-SVM adaptively selects reasonable parameters during training, so the classification performance is improved and shows better generalization and fitting ability on the same test set.

## 5. Conclusions

This paper proposes a multi-type concrete defect non-destructive classification algorithm, realizing non-contact concrete defect signal detection and automatic classification by an SVM and laser vibrometry technology. An ultrasonic transducer was used to excite concrete specimens with different defects, and then an LDV was used to acquire the vibration signals on their surfaces. The original detection signal was decomposed into four layers by wavelet packet transform. Three time-frequency features, nodal energy ratio variation rate, nodal energy ratio, and wavelet packet singular entropy, were obtained to classify and identify the detection signal by SVM. The MSSMA algorithm is proposed to optimize the SVM parameters for the learning performance of the model. Four-classifying experiments on 520 detection data samples were carried out, constituting four datasets with no defects, segregation, foreign matter, and cavity. The experimental results show that the proposed algorithm effectively classifies the ultrasonic defect signals of c30-class concrete measured by laser vibrometry and improves the accuracy of classification results on previous traditional machine learning methods.

In future work, we will continue to optimize the algorithm model and investigate the classification performance of the algorithm for concrete defects with different ultrasonic frequency excitation and sizes, In addition, testing the classification effect of our algorithm on various test blocks is also an essential task.

## Figures and Tables

**Table 1:** The classification results of different layers.

Level	Data1	Data2	Data3	Data4	Total
L2	75.00%	13.33%	15.00%	10.00%	28.33%
L3	91.67%	5.00%	21.67%	8.33%	31.68%
L4	83.33%	91.67%	96.67%	91.67%	90.83%
L5	96.67%	66.67%	96.67%	58.33%	79.58%

**Table 2:** The classification results of different features.

Feature	Data1	Data2	Data3	Data4	Total
feature-1	21.67%	12.67%	36.67%	66.67%	34.17%
feature-2	78.83%	88.33%	95.00%	91.67%	88.83%
feature-3	71.67%	90.00%	90.00%	73.33%	81.25%
feature-all	83.33%	91.67%	96.67%	91.67%	90.83%

**Table 3:** Classification results of different models.

Model	Values	Data1	Data2	Data3	Data4	Total
SVM	AVG	88.33%	91.67%	96.67%	91.67%	90.83%
	STD	\	\	\	\	\
GWO-SVM	AVG	95.43%	90.70%	95.60%	93.47%	93.80%
	STD	3.80	3.96	1.57	4.71	3.50
WOA-SVM	AVG	92.63%	87.83%	94.70%	90.07%	91.31%
	STD	4.18	3.99	1.53	4.78	3.58
MFO-SVM	AVG	94.67%	90.22%	95.33%	91.89%	93.03%
	STD	4.45	5.32	1.69	5.07	3.74
SMA-SVM	AVG	96.20%	93.43%	95.53%	94.47%	94.91%
	STD	7.81	1.59	6.45	7.24	5.25
MSSMA-SVM	AVG	98.13%	93.67%	96.67%	96.67%	96.29%
	STD	0.74	0.85	0.02	0.04	0.18

**Table 4:** Parameter setting of each algorithm.

Algorithm	Parameter Setting
BP	training times = 500; goal = 0.005; learning rate = 0.01
RF	ntree = 200; mtry = 2
SCN	hidden layer nodes = 50; learning rate = 0.01
	maximum number of random configurations = 100,
	random weight range = [0.5, 1, 5, 10, 30, 50, 100],
	inequality constraint coefficient = [0.9, 0.99, 0.999, 0.9999, 0.99999, 0.999999]

**Table 5:** The average accuracy using different algorithms.

Model	Values	Data1	Data2	Data3	Data4	Total
BP	AVG	89.54%	96.04%	96.59%	82.13%	91.08%
RF	AVG	81.99%	95.59%	95.15%	97.47%	92.55%
SCN	AVG	89.60%	91.20%	91.23%	88.17%	90.05%
MSSMA-SVM	AVG	98.13%	93.67%	96.67%	96.67%	96.29%

## Data Availability

The data sets in this study are available on request from the corresponding author. The data is not publicly available due to privacy concerns.
